# Sevestre-Jacquet Posterosive Syphilitic Dermatitis: A Case Report

**DOI:** 10.7759/cureus.98846

**Published:** 2025-12-09

**Authors:** Jorge Alberto Cortez Vila, Carlos Josue Arellanes Guillen, José E Pérez-Nieto, Luisa Mariana Guerrero Escudero, Cristina Berumen-Glinz, Elisa Vega-Memije

**Affiliations:** 1 Department of Dermatology, Hospital General Dr. Manuel Gea González, Mexico City, MEX

**Keywords:** cutaneous, diaper rash, skin diseases, syphilis, urinary incontinence

## Abstract

Diaper area dermatitis is a common inflammatory reaction in infants, although it may occur at other ages. One of its severe forms is Sevestre-Jacquet posterosive syphilitic dermatitis, characterized by ulcerated papulo-nodular lesions in areas exposed to urine or feces. We present an atypical case in a nine-year-old boy, an unusual age for the onset of this entity, which is typically seen in infants and young children who use diapers. Initially, child sexual abuse was suspected; however, the diagnosis was confirmed through histopathological examination. This type of dermatitis poses diagnostic challenges when it occurs outside its classic context, warranting careful clinical assessment and complementary studies for proper differentiation.

## Introduction

Diaper dermatitis (DD) is a general term describing an inflammatory skin reaction in the diaper area. In infants, the estimated prevalence of diaper dermatitis ranges from 7 to over 40% [1]. Although it can occur at any age, it is most commonly seen in newborns and infants under two years old. The etiology of DD is complex. Irritation due to activation of fecal enzymes, increase of skin pH, prolonged exposure to moisture, and mechanical factors is responsible for irritant contact dermatitis [2]. Mild diaper rash may present with mild erythema, scattered erythematous papules and itching. Severe forms may present with extensive erythema with maceration and erosions [1]. Among the spectrum of diaper dermatitis, irritant contact dermatitis is the most frequent form. In its more severe presentations, various entities may arise, including Sevestre-Jacquet posterosive syphilid dermatitis. This condition has been associated with poor hygiene, diarrhea, genetic susceptibility, detergent residues, and the use of cloth diapers. The classic clinical presentation includes ulcerated or eroded papulo-nodular lesions on an erythematous base [2,3].

We describe an uncommon presentation of Sevestre-Jacquet posterosive syphilitic dermatitis in a child with incontinence, with no identifiable association to diaper use.

## Case presentation

A nine-year-old male patient, with no significant medical history and complete vaccination schedule, was brought to the pediatric emergency department by his parents due to a genital dermatosis noted a week earlier during bathing. Upon direct questioning, the patient reported a one-month evolution of the lesions following a trip to the beach.

Primary care physicians noted inconsistencies in the parents’ history, leading to an initial presumptive diagnosis of Kempe syndrome and suspected sexual abuse. The patient was admitted to the pediatric ward, and empirical treatment was initiated with ceftriaxone 1 g every 24 hours, clarithromycin 250 mg every eight hours, and acyclovir 800 mg every eight hours, for a total duration of one week.

**Figure 1:**
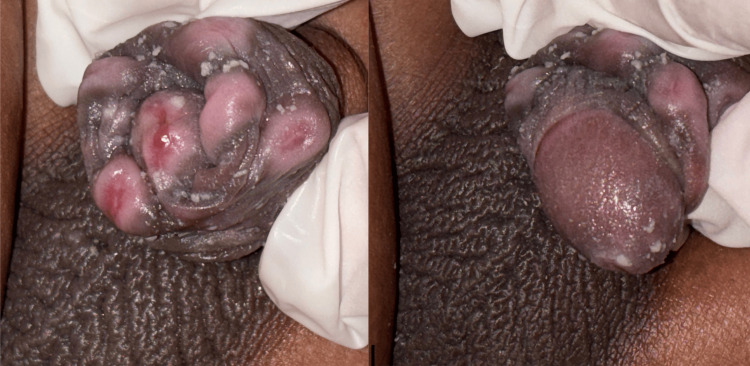
Dermatosis in the genital region involving the foreskin. Six well-defined, non-confluent, indurated, ulcerated papules with a pink-violet hue were noted.

Subsequent dermatology evaluation revealed a localized dermatosis in the genital region, affecting the foreskin. Six well-defined, non-confluent, indurated, ulcerated papules of pink-violet coloration were observed (Figure 1). Poor local hygiene with the presence of smegma was noted, along with bilateral cryptorchidism. No lymphadenopathy was identified.

On psychosocial evaluation, the patient appeared introverted, with signs of selective mutism, enuresis, nocturia, and encopresis. He reported a history of school bullying and prolonged video game use.

Laboratory tests were performed, all within normal parameters for the patient’s sex and height. 

A screening panel for sexually transmitted infections was requested, which yielded negative results for venereal disease research laboratory (VDRL) test, human immunodeficiency virus (HIV), hepatitis B virus (HBV), hepatitis C virus (HCV), and herpes simplex virus (HSV); however, fluorescent treponemal antibody absorption (FTA-ABS) test results were still pending.

**Figure 2:**
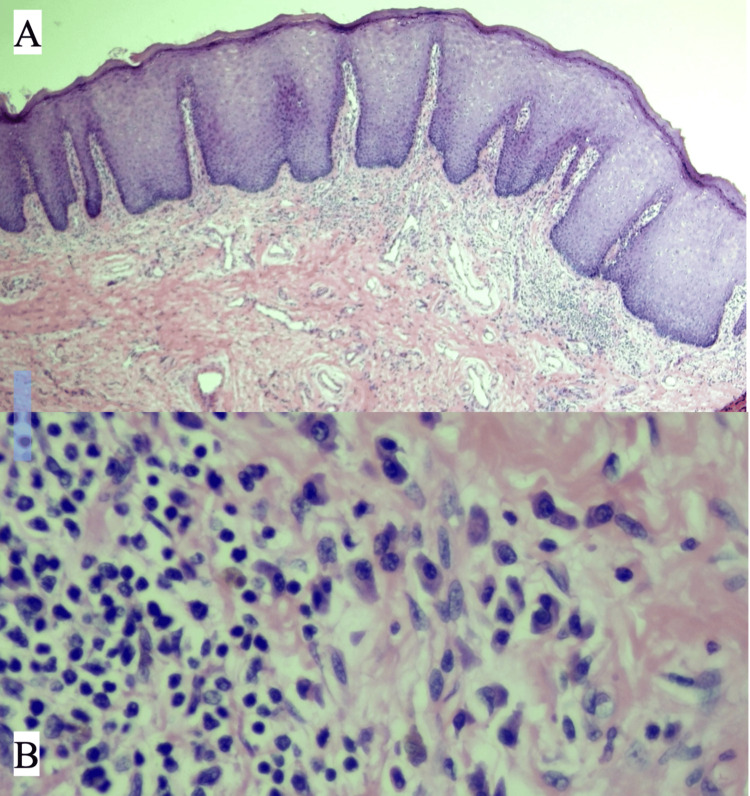
Skin biopsy stained with H&E (A) Regular acanthosis, hypergranulosis, clear cells, and a band-like inflammatory infiltrate in the papillary dermis. (B) The infiltrate consists of plasma cells, lymphocytes, and histiocytes.

A biopsy of one of the lesions was performed, revealing findings of regular acanthosis, hypergranulosis, clear cells, and a band-like inflammatory infiltrate in the papillary dermis composed of plasma cells, lymphocytes, and histiocytes (Figure 2). Two days later, the urology team performed an orchidopexy to correct bilateral cryptorchidism, along with circumcision. Tissue was sent to the pathology department, and histologic findings were consistent with those of the initial biopsy.

Immunohistochemistry for *Treponema pallidum* and universal human papillomavirus polymerase chain reaction (HPV PCR) testing were both negative. Based on these histopathological findings, the diagnosis of Sevestre-Jacquet posterosive syphilitic dermatitis was confirmed.

Antibiotic therapy was discontinued, and the patient was discharged. A pediatric psychiatry consultation ruled out both Kempe syndrome and sexual abuse.

**Figure 3:**
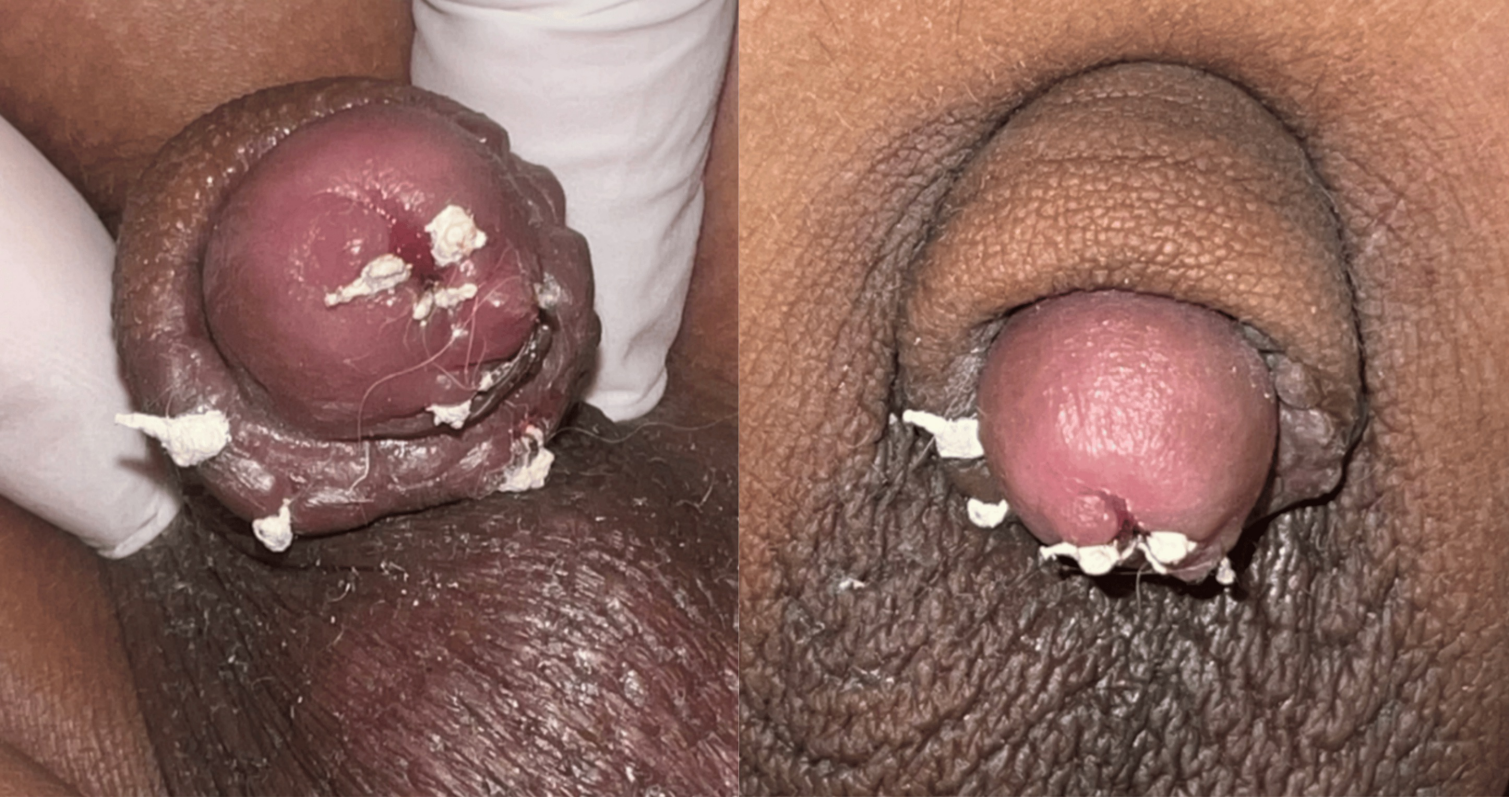
Clinical images taken three weeks after circumcision No new lesions were observed.

At a follow-up dermatology visit three weeks after discharge, appropriate healing and no new lesions were observed (Figure 3). A new FTA-ABS test was also requested and returned negative.

## Discussion

The present case corresponds to a posterosive syphilid dermatitis of Sevestre-Jacquet; however, as we will discuss below, it presents features that make it atypical. One of the most relevant aspects is that the patient was neither an infant nor used diapers, which excludes the classic context in which these lesions are typically observed. In addition, the presence of conflicting elements in the medical history complicated the initial diagnostic approach.

Posterosive syphilitic dermatitis, also known as Jacquet’s erosive diaper dermatitis, is an uncommon condition that typically affects infants but may also occur in adults with fecal or urinary incontinence. It is considered a non-treponemal, syphilid-like dermatitis, historically named for its resemblance to syphilitic lesions. Macroscopically, it is characterized by purplish-red, well-demarcated lesions with umbilicated centers, usually measuring 2-5 mm in diameter, located on the perianal or genital skin. These lesions often progress to superficial erosions or ulcers [4].

It is important to consider incontinence-associated dermatitis (IAD), a condition that falls within the spectrum of moisture-associated skin damage (MASD). Its pathogenesis involves maceration due to prolonged contact with urine, resulting in thinning of the epidermis, altered blood flow, and decreased skin pH. Additional damage is caused by fecal proteases and colonization by commensal microorganisms, particularly coliform bacteria and *Candida spp.* [5]. It has been proposed that this condition may be grouped with diaper dermatitis due to their shared pathophysiology; however, given their distinct definitions and clinical presentations, they should still be considered as separate entities [6].

Considering that Sevestre-Jacquet posterosive dermatitis is not exclusive to diaper users, it may occur at different ages and with variable clinical forms. Atypical cases have been documented in the literature and have been associated with incontinence-associated dermatitis. Amin et al. reported the case of a 21-year-old woman with chronic umbilical drainage and a three-year history of a periumbilical eruption characterized by red, friable, well-demarcated papules [7]. Hara et al. described a nine-year-old girl with urinary incontinence due to an ectopic opening of a duplicated left ureter into the vaginal vestibule. The use of toilet paper as an absorbent was considered one of the contributing factors to her rash. The lesions resolved with topical treatment using a nonsteroidal anti-inflammatory ointment and zinc oxide ointment, along with the use of sanitary pads [8]. Therefore, we propose that posterosive syphilid dermatitis may appear in both clinical contexts.

**Figure 4:**
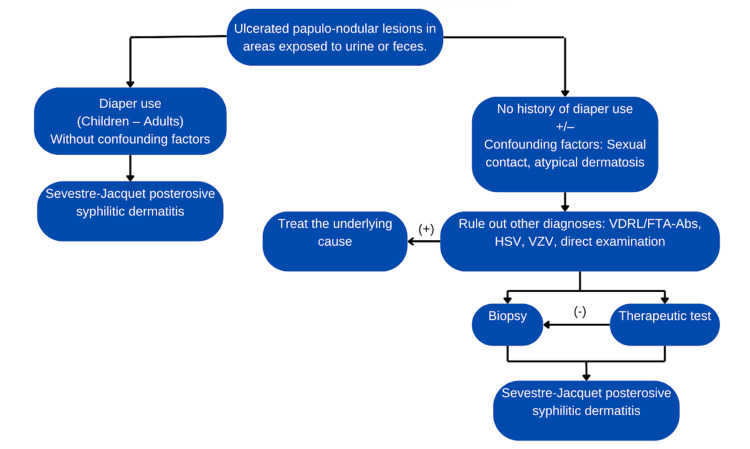
Diagnostic algorithm VDRL: venereal disease research laboratory, FTA-Abs: fluorescent treponemal antibody absorption, HSV: herpes simplex virus, VZV: varicella zoster virus, Image Credits: Jorge Alberto Cortez Vila

We suggest the following algorithm for the diagnosis of ulcerated-erosive papulo-nodular lesions in areas exposed to urine or feces (Figure 4).

On one hand, there are patients with a clear history of diaper use and a consistent clinical history without confounding factors. In these cases, the diagnosis of Sevestre-Jacquet posterosive syphilid dermatitis can be made by exclusion, supported by clinical findings and disease progression.

On the other hand, we may encounter patients with no history of diaper use and the presence of confounding factors such as sexual contact, atopic dermatitis, or some form of incontinence, such as enuresis. In such cases, it is essential to rule out other diagnoses. If these are negative, a skin biopsy or therapeutic diagnostic trial may be considered.

The skin biopsy of this entity shows epidermal changes such as hyperplasia, spongiosis, and acanthosis, along with a nonspecific inflammatory infiltrate with lymphocytes. Because of its topography, it may also show a significant number of plasma cells-a common finding that can be mistaken for secondary syphilis. Therefore, the use of non-treponemal tests, such as VDRL, is necessary to rule out this condition.

Although the diagnosis of Sevestre-Jacquet erosive diaper dermatitis is typically based on clinical history and cutaneous manifestations, it is important to consider differential diagnoses such as perianal pseudoverrucous papules and nodules (PPPN) and infantile gluteal granuloma, as they share similar histological features. Other potential causes of diaper area dermatitis should also be considered (Table 1).

**Table 1:** Other potential causes of diaper area dermatitis Table Credits: Jorge Alberto Cortez Vila

Differential diagnoses
Congenital / acquired syphilis – Abuse
Herpes simplex
Atypical molluscum contagiosum
Human papillomavirus
Candidiasis
Allergic contact dermatitis
Langerhans cell histiocytosis
Seborrheic dermatitis
Atopic dermatitis
Psoriasis

General management is the cornerstone of treatment. It includes frequent diaper changes to reduce prolonged exposure to urine and feces, as well as allowing brief periods of air exposure to keep the area dry. Regarding topical treatment, preparations containing barrier agents such as zinc oxide help protect the skin from chemical irritants and reduce friction. If dermatitis persists, low-potency topical corticosteroids may be considered, avoiding fluorinated agents due to the risk of systemic absorption. In cases of secondary bacterial infection caused by gram-positive cocci, mupirocin or fusidic acid may be added. If Candida superinfection is suspected, antifungals such as nystatin or clotrimazole are indicated, although these may cause local side effects such as irritation or burning [1].

In the presented case, circumcision was considered both diagnostic and therapeutic.

## Conclusions

We described a severe form of diaper dermatitis in a child associated with incontinence, with a definitive histopathological diagnosis of Sevestre-Jacquet posterosive syphilid dermatitis. Current descriptions and definitions create ambiguity in diagnosis, making it necessary to consider diaper dermatitis and incontinence-associated dermatitis as distinct entities. Posterosive syphilid dermatitis may occur in the context of either.

In patients without a history of diaper use and with confounding factors, this condition poses a significant diagnostic challenge, especially due to its clinical similarity to sexually transmitted infections.
